# Monolithic Perovskite/Silicon Tandem Solar Cells Enabled by Multifunctional TiO_x_ Interconnects

**DOI:** 10.1002/smll.202500969

**Published:** 2025-04-27

**Authors:** Takuya Matsui, Calum McDonald, Abduheber Mirzehmet, James McQueen, Ruy Sebastian Bonilla, Hitoshi Sai

**Affiliations:** ^1^ Renewable Energy Advanced Research Center National Institute of Advanced Industrial Science and Technology (AIST) 1‐1‐1 Umezono Tsukuba Ibaraki 305‐8568 Japan; ^2^ Department of Materials University of Oxford 16 Parks Rd Oxford OX1 3PH UK

**Keywords:** passivating contact, perovskite/silicon tandem solar cell, recombination junction, titanium nitride, titanium oxide

## Abstract

Perovskite‐on‐silicon tandem solar cells have emerged as a leading technology enabling high power conversion efficiencies (PCE) over 30%. Despite current progress, the intrinsic multilayer device design presents vast challenges in complexity, which can be a drawback in future mass production. Multifunctional nanolayer materials that simplify large‐scale production are therefore highly desirable. Herein, a TiO_x_ layer (∼3–5 nm) grown by atomic layer deposition (ALD) enables a series interconnection of a perovskite *n‐i‐p* top cell with a silicon wafer directly. The TiO_x_ layer serves as an *all‐in‐one* interconnect, fulfilling the functions of silicon surface passivation, hole extraction from silicon, and recombination junction at the top/bottom cell interface. As a result, a proof‐of‐concept 22.4%‐efficient tandem device is demonstrated. Furthermore, an improved PCE of 26.5% is achieved by capping the TiO_x_ with a thin ALD‐TiN_y_ layer (∼4 nm). This represents a performance as high as the reference tandem device (PCE = 25.5%) that uses amorphous silicon passivating selective contacts and an indium‐tin‐oxide interlayer. Such a TiO_x_ multifunctional nanolayer can reduce the number of deposition tools and eliminate the need of an indium‐based interconnection. It offers a potential for low‐cost, scalable, and sustainable tandem solar cell manufacturing.

## Graphical abstract

TiO_x_ nanolayer enables a series interconnection of a perovskite top cell with a silicon wafer directly in perovskite/silicon tandem solar cells. The TiO_x_ serves as an *all‐in‐one* interconnect, providing surface passivation, hole transport, and recombination junction. A TiN_y_ capping layer boosts performance to a level comparable to that of conventional tandem devices employing amorphous silicon and indium oxide layers.

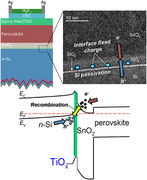

## 1 Introduction

Crystalline Si solar cells, which account for over 95% of the global market today,^[^ ^1^ ^]^ require innovative breakthroughs to further enhance efficiency while maintaining cost‐effectiveness and sustainable manufacturing processes, enabling multi‐terawatt scale production.^[^ ^2^ ^]^ The power conversion efficiency (PCE) of the state‐of‐the‐art Si solar cells (∼27%)^[^ ^3^ ^]^ is approaching its theoretical limit of 29.4%,^[^ ^4^ ^]^ making it impossible to achieve higher PCEs with Si alone. Recently, a perovskite/Si tandem solar cell architecture has gathered attention to surpass the limitations of Si. Small‐area laboratory devices (∼1 cm^2^) have been reported with PCEs over 33%,^[^ ^5^, ^6^ ^]^ which is well above the PCEs of Si and perovskite single‐junction devices.

From an industrial perspective, however, the trend toward increasing the number of layers stacked to attain higher tandem cell efficiency poses critical challenges in scalability and deployment. The most prevalent Si bottom cell used in perovskite/Si tandem devices is the silicon heterojunction (SHJ) architecture,^[^ ^5^, ^6^, ^7^, ^8^, ^9^, ^10^, ^11^, ^12^, ^13^ ^]^ which uses hydrogenated amorphous Si (a‐Si:H) thin layers at the front and rear of the Si wafer. Despite their broad use, the global market share of SHJ technology is still below 10%^[^ ^1^ ^]^ due to the higher capital expenditure and material cost (indium and silver) compared to the Si homojunction architecture, including passivated emitter rear cell (PERC)^[^ ^14^, ^15^ ^]^ and tunnel oxide passivated contact (TOPCon).^[^ ^16^, ^17^ ^]^ Furthermore, the recombination junction (RJ) (p/n inverse junction), which is key to interconnect the perovskite top cell and Si bottom cell, is mostly formed using indium‐based oxides such as an indium‐tin oxide (ITO) made by physical vapor deposition. This leads to a higher consumption of indium which is a critical and scarce metal.^[^ ^18^ ^]^

We have recently developed a titanium oxide (TiO_x_ or TiO_2_) nanolayer grown by atomic layer deposition (ALD) that uniquely acts as a hole‐selective passivating contact in Si solar cells.^[^ ^19^, ^20^ ^]^ We further demonstrated that the carrier selectivity of the TiO_x_ can be tailored by the ALD process^[^ ^21^ ^]^ and/or the choice of Ti precursors.^[^ ^22^ ^]^ The hole selective function in TiO_x_ is in contrast to the generally understood behavior of TiO_x_. TiO_x_ is typically considered an electron selective material with respect to various solar cell absorbers including Si,^[^ ^23^, ^24^ ^]^ III‐V materials such as InP^[^ ^25^ ^]^ and perovskite.^[^ ^26^, ^27^ ^]^ Using our TiO_x_ nanolayer (∼5 nm) as a hole contact at the front or rear of both *n*‐ and *p*‐type Si, PCEs of >20% have been demonstrated without using any buffer layer between the Si absorber and TiO_x_ contact layer.^[^ ^19^, ^20^ ^]^ This unique function of the TiO_x_ is particularly suited for its application to homojunction solar cells because it offers the potential to replace the dielectric insulator layers (such as Al_2_O_3_ and Si_3_N_4_) with the semiconducting TiO_x_ passivating contact. It also facilitates 1D charge carrier transport from the Si absorber to the contacts, without resulting in unpassivated Si‐metal local interfaces. We now take this material to the next level by demonstrating that this technology can be applied as an interconnection between Si and perovskite solar cells in a tandem architecture.

In this work, we apply our hole‐selective TiO_x_ passivating contact in perovskite/Si tandem solar cells. While ALD‐TiO_x_ has been previously used to form an RJ in perovskite/Si tandem solar cells,^[^ ^28^ ^]^ our new TiO_x_ concept reported in this work not only forms an RJ with the charge transport layer of the top cell, but also performs the surface passivation and the hole transport layer of the Si bottom cell. Furthermore, we show that the TiO_x_ layer deposition followed by an ALD‐TiN_y_ capping can boost the tandem cell efficiency to be comparable with the tandem devices that use standard SHJ bottom cells and ITO interlayers. This multifunctionality of our TiO_x_ allows us to reduce the number of deposition processes, simplifying the tandem structure without using indium‐based interconnection materials. Finally, we propose that this TiO_x_ technology enables the potential of integrating industrial homojunction solar cells (like PERC and TOPCon) into a tandem architecture.

## 2 Results and Discussion

**Figure 1:**
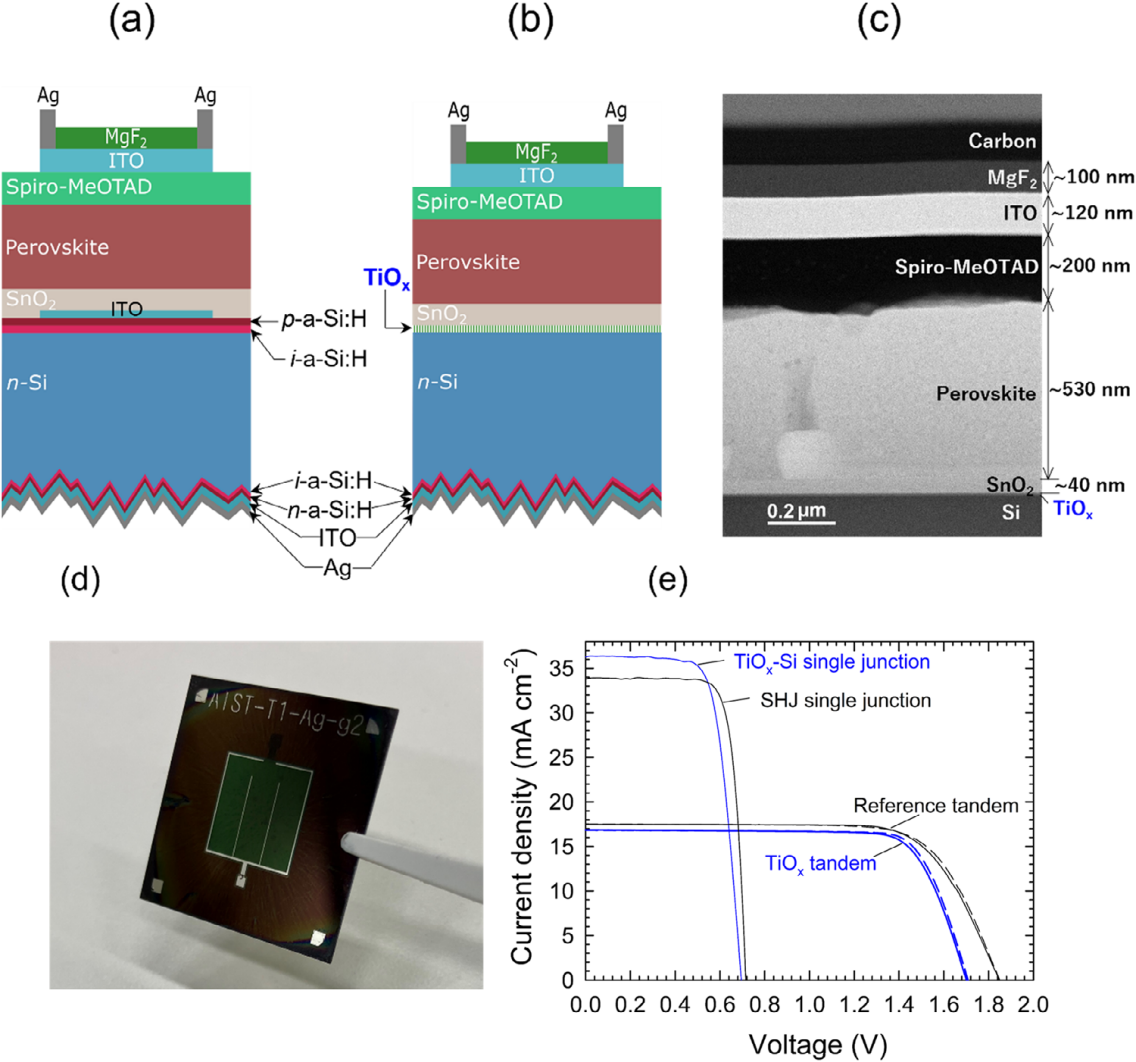
Tandem device architecture and performance. a,b) Schematic illustrations of solar cells fabricated in this work ((a) reference tandem and (b) TiO_x_ tandem). c) Cross‐sectional high‐annular angle dark‐field scanning transmission electron microscopy (HAADF‐STEM) image of the perovskite top cell (TiO_x_ tandem). d) A photo of the tandem solar cell fabricated on a 25 mm × 25 mm Si substrate. e) J‐V curves of the reference tandem (black lines) and the TiO_x_ tandem (blue lines). The full and dashed lines represent the J‐V curves measured in forward and backward scans, respectively. The J‐V curves of the corresponding Si single‐junction solar cells are also shown (black: rear contact/*n*‐Si/*i*‐a‐Si:H/*p*‐a‐Si:H/ITO/Ag‐grid (SHJ), blue: rear contact/*n*‐Si/TiO_x_/ITO/Ag‐grid (TiO_x_‐Si cell). The detailed information of these single‐junction solar cells is given in Figures S1 and S2, Supporting Information).

**Figure** **1**a,b shows the schematic diagrams of perovskite/Si tandem devices fabricated in this study. The device in Figure 1a is based on a standard SHJ bottom cell architecture used as a reference, while that shown in Figure 1b features TiO_x_ layer deposited on the front side of *n*‐Si wafer. For both devices, front‐planar and rear‐textured *n*‐Si wafers were used, and the rear side was made with an electron contact composed of an *i‐n* a‐Si:H/ITO/Ag layer stack. These tandem devices have a perovskite top cell made entirely by solution process (spin coating) in an *n‐i‐p* deposition sequence. The perovskite top cell consists of SnO_2_ (∼40 nm) as electron transport layer (ETL), perovskite absorber (∼530 nm) with a bandgap of 1.63 eV, and doped 2,2′,7,7′‐Tetrakis[N,N‐di(4‐methoxyphenyl)amino]‐9,9′‐spirobifluorene (Spiro‐MeOTAD) (∼200 nm) as hole transport layer (HTL). An ITO front electrode was formed by sputtering followed by Ag‐grid deposition by either sputtering or thermal evaporation, and an MgF_2_ antireflection layer was formed by thermal evaporation. The tandem cell shown in Figure 1a has an ITO interlayer to form an RJ at the interface between top and bottom cells. On the other hand, for the device shown in Figure 1b, the hole‐selective TiO_x_ layer (emitter) was deposited directly on an *n*‐Si wafer by thermal ALD,^[^ ^19^, ^22^ ^]^ which is interconnected by the ETL of the top cell. Hereafter, we term these devices “reference tandem” and “TiO_x_ tandem”, respectively. A cross‐sectional transmission electron microscopy (TEM) image of the TiO_x_ tandem is shown in Figure 1c, where the thicknesses of the perovskite top‐cell component layers can be identified. A photo of tandem device fabricated on a 25 mm × 25 mm Si substrate is shown in Figure 1d. With a black shadow mask (not shown), the illumination area is designated to 1.0 cm^2^. The current density‐voltage (J‐V) curves of the reference tandem and the TiO_x_ tandem are presented in Figure 1e. It has been reported that, without an ITO interlayer in the reference tandem, the J‐V results in an S‐shape curve due to a non‐ideal Schottky barrier developed between the top and bottom cells.^[^ ^29^ ^]^ This is a well‐known difficulty in contacting the carrier selective contact of the Si bottom cell to the charge transport layer of the perovskite top cell. Nevertheless, the TiO_x_ tandem exhibits a diode J‐V curve without using such an additional interlayer, as shown in Figure 1e (blue lines).

Despite such a simple device structure, the TiO_x_ tandem exhibits a PCE of 22.4% (short‐circuit current density (J_SC_) = 16.8 mA cm^−2^, open‐circuit voltage (V_OC_) = 1.704 V, fill factor (FF) = 0.784) (blue dashed line in Figure 1e). The V_OC_ of this device is well above the V_OC_ of the Si (∼0.7 V) and perovskite (∼1.15–1.2 V) single‐junction solar cells. More detailed information about the device structures and properties of these single‐junction solar cells is provided in Figures S1 and S2 (Supporting Information). To the best of our knowledge, this is the first demonstration of a perovskite/Si tandem device whose subcells are interconnected through a non‐Si passivation contact of the bottom cell. The experimental result of the TiO_x_ tandem demonstrates that a 5‐nm‐thick TiO_x_ nanolayer provides *all‐in‐one* functionality: Si surface passivation, hole extraction from Si, and RJ at the top/bottom cell interface. However, the reference tandem that comprises three layers (i.e., *i*‐a‐Si:H/*p*‐a‐Si:H/ITO) for fulfilling each function shows a higher PCE of 23.8% (J_SC_ = 17.3 mA cm^−2^, V_OC_ = 1.837 V, FF = 0.748) (black dashed line in Figure 1e). The V_OC_ of this device is nearly equivalent to the sum of the V_OC_ of each subcell. The lower V_OC_ of the TiO_x_ tandem can be attributed in part to the lower V_OC_ of the Si bottom cell (Figure 1e). However, the V_OC_ of these single‐junction devices differs only by 20 mV, which cannot fully account for the V_OC_ loss of ∼>150 mV observed in the tandem devices. The mechanism behind this V_OC_ loss and a way of mitigating it will be provided in the following sections. Meanwhile, the slightly lower J_SC_ of the TiO_x_ tandem compared to that of the reference tandem (Figure 1e) is caused by the batch‐to‐batch variation of thicknesses of component layers (mainly the HTL thickness) that results in the different current matching conditions. The difference in J_SC_ of single‐junction devices originates from the lower parasitic absorption loss in the short wavelengths for TiO_x_ compared to a‐Si:H layers (Figures S1, Supporting Information). However, this effect is not reflected in tandem cells because the short wavelength light is completely absorbed by the top cell.

**Figure 2:**
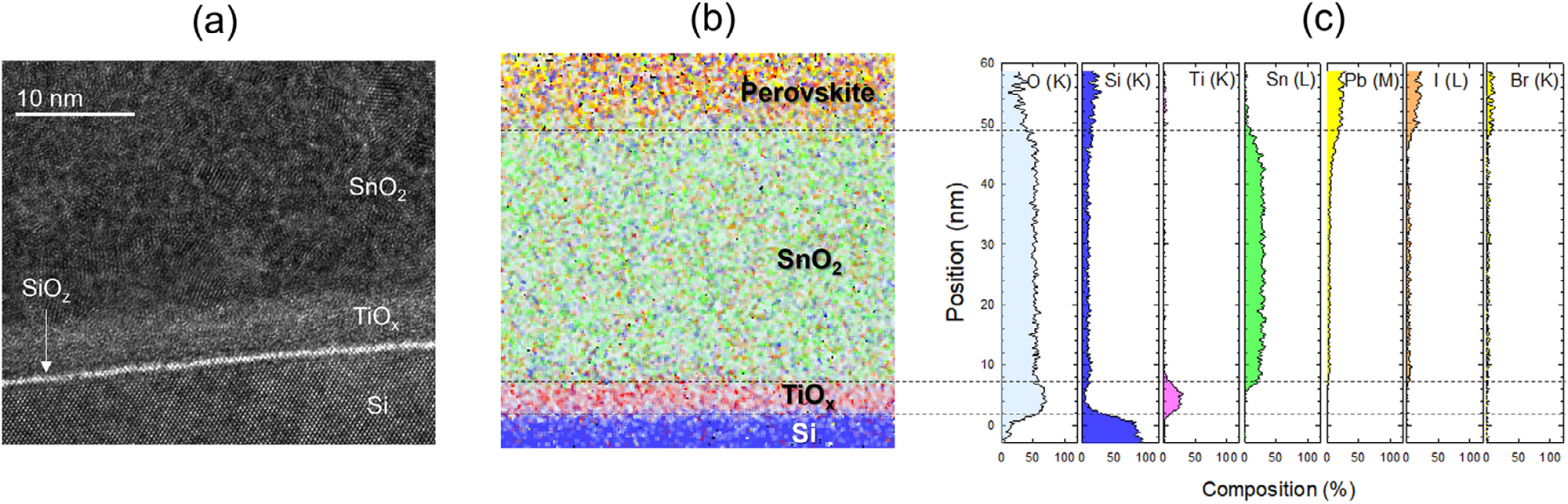
Top/bottom interface structure. a) Cross‐sectional bright‐field transmission electron microscope (BF‐TEM) image at the top/bottom cell interface of the TiO_x_ tandem, and its b) EDX elemental map and c) line profiles.

**Figure** **2**a shows the bright‐field transmission electron microscope (BF‐TEM) image taken at the cross‐section of the top/bottom cell interface of the TiO_x_ tandem. The elemental map and its line profiles measured by energy dispersive x‐ray (EDX) analysis are shown in Figure 2b,c, respectively. Both TEM image and EDX map show atomically sharp Si/TiO_x_ and TiO_x_/SnO_2_ interfaces. Similar to previous studies,^[^ ^22^, ^30^ ^]^ a ∼1 nm‐thick SiO_z_ (SiO_2_) interlayer that contains a slight amount of Ti is spontaneously created at the Si/TiO_x_ interface. The ∼5‐nm‐thick TiO_x_ layer exhibits a predominantly amorphous microstructure since no lattice fringe pattern is identified in the TEM image. This amorphous nature differs strikingly from the ALD‐TiO_x_ layer previously used in the perovskite/Si tandem solar cells.^[^ ^28^ ^]^ In addition, this amorphous structure of the TiO_x_ layer contrasts with the nanocrystalline structure observed in the spin‐coated SnO_2_ layer, where lattice fringe patterns with <10 nm diameter are visible. Interestingly, the EDX line profiles indicate that the elements of the perovskite absorber layer (such as Pb, I, and Br) penetrate into the SnO_2_ layer. The migration of halide atoms into SnO_2_ and even underlying ITO layer has been reported in single‐junction devices and it is responsible for the performance degradation.^[^ ^31^ ^]^ In Figure 2c, however, there is a sudden drop off in the Pb and halide signal at the interface with TiO_x_, indicating that Pb and halide species cannot penetrate the TiO_x_ layer probably due to its denser amorphous nature. Thus, we highlight that the TiO_x_ layer provides an additional function of protecting Si bottom cell from chemical reactions arising from the in‐diffusion of lead and highly reactive halide species.

**Figure 3:**
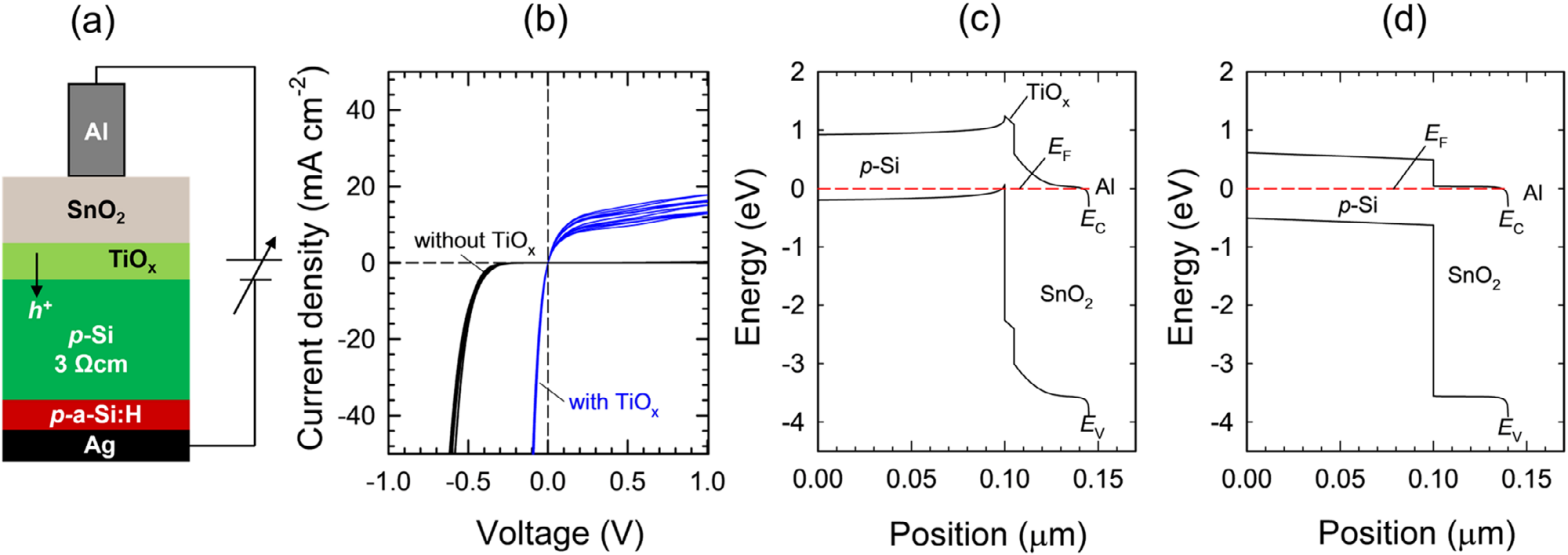
Current flow mechanism at the recombination junction. a) Test structure for contact resistivity measurement and b) the corresponding dark J‐V characteristics with and without a TiO_x_ layer between the Si wafer and the SnO_2_ layer. Six identical Al pads were fabricated on a substrate and measured with both forward and backward voltage scans. Note that *p*‐Si was used as substrate instead of *n*‐Si to characterize the hole injection from Si to TiO_x_/SnO_2_ recombination junction. c,d) Band diagrams of the test structures c) with TiO_x_ and d) without TiO_x_ layer obtained by a SCAPS finite element device simulation. *E*_C_ and *E*_V_ denote energy levels of conduction band and valence band, respectively. *E*_F_ is the Fermi level.

We next investigate the electrical contact properties of the TiO_x_ layer with a focus on its role as an RJ in the tandem device. We prepared specific test samples consisting of the same layers as used in the tandem devices and measured the dark J‐V characteristics, as shown in **Figure** **3**a. Note that *p*‐Si was used instead of *n*‐Si to study the degree of recombination of majority carriers injected from Si (holes) and SnO_2_ (electrons) at the RJ. Al was chosen to have a good electrical contact with the SnO_2_ layer. As shown in Figure 3b, the dark J‐V curves exhibit a rectifying behavior, while the threshold voltage is largely influenced by the presence of the TiO_x_ layer. When the TiO_x_ layer is present between the *p*‐Si and the SnO_2_ layer, high current flows even when a very low reverse voltage is applied (J = 20 mA cm^−2^ at V ∼ 0.05 V). The similar level of contact resistivity was measured for the *p*‐Si/TiO_x_ interface.^[^ ^22^ ^]^ However, the test sample without a TiO_x_ layer requires a higher voltage (V ∼ 0.5 V) to achieve the same current density. In the forward‐biased regime, much lower current flows particularly for the sample without the TiO_x_ layer. To explain these results, device simulations were carried out for the same sample structures using the SCAPS_1D finite element device simulator.^[^ ^32^ ^]^ The band diagrams obtained by the simulation with and without the TiO_x_ layer are shown in Figure 3c,d, respectively. Here, we assume a presence of fixed negative charges at the *p*‐Si/TiO_x_ interface in the order of 10^13^ cm^−2^, based on the band bending measurement for the *n*‐Si/TiO_x_ interface.^[^ ^22^ ^]^ The detailed parameters assumed in the simulation are given in Table S1 (Supporting Information). In Figure 3c, hole accumulation occurs at the *p*‐Si/TiO_x_ interface due to the presence of a high concentration of fixed negative charge. In an actual tandem device, a hole inversion layer is induced in *n*‐Si near the *n*‐Si/TiO_x_ interface.^[^ ^22^ ^]^ This facilitates the transport of holes in the Si wafer to the SnO_2_ layer through the trap‐assisted tunneling mechanism, within the bandgap of the TiO_x_ layer, allowing the current flow by the exchange of majority carriers.^[^ ^33^ ^]^ The lesser voltage dependence observed in the forward bias regime indicates tunneling of electrons in the valence band of *p*‐Si to SnO_2_, which is similar to the J‐V behavior of a metal‐insulator‐semiconductor tunnel diode when the semiconductor is in accumulation mode.^[^ ^34^, ^35^ ^]^ In contrast, the band diagram without the TiO_x_ layer (Figure 3d) indicates the depletion of holes in the *p*‐Si near the *p*‐Si/SnO_2_ interface due to the work function difference between these two materials. This situation hinders the majority carrier recombination and thus the current flow without applying a large bias voltage equivalent to the conduction band offset at the *p*‐Si/SnO_2_ interface (∼0.5 eV), in agreement with the experimental J‐V curves. These simulation results account for the significantly different dark J‐V behaviors depending on the presence of the TiO_x_ layer between the Si and the ETL (SnO_2_) of the top cell.

Although the TiO_x_ monolayer can provide multiple functions, one major issue in replacing the conventional thin multilayers with the TiO_x_ monolayer is a non‐negligible V_OC_ loss of ∼>150 mV (Figure 1e). We confirmed that this V_OC_ loss is markedly larger than that of the Si single‐junction solar cells (∼20 mV). To address this issue, we have introduced an ALD‐TiN_y_ capping layer on top of the TiO_x_ layer. TiN_y_ was chosen because of its relatively high work function (4.40–4.53 eV),^[^ ^37^ ^]^ since the passivation and carrier selectivity of the TiO_x_ layer depend on the work function of the capping material.^[^ ^20^, ^21^, ^22^, ^36^ ^]^ In addition, TiN_y_ is known to exhibit relatively high electrical conductivity arising from its ease of partial crystallization (Figure S3, Supporting Information). The TiN_y_ layer was deposited by thermal ALD using tetrakis(dimethylamido)titanium (TDMAT) as the Ti precursor and NH₃ as the reactant gas. Because the particular ALD system used for TiO_x_ deposition did not support NH₃ gas, the TiN_y_ layer was deposited in a separate ALD system. However, in principle, the TiN_y_ layer can also be deposited in the same ALD chamber immediately after the TiO_x_ layer deposition and the following hydrogen plasma treatment.^[^ ^19^ ^]^ This enables the integration of a TiO_x_/TiN_y_ stack using a single deposition tool.

**Figure 4:**
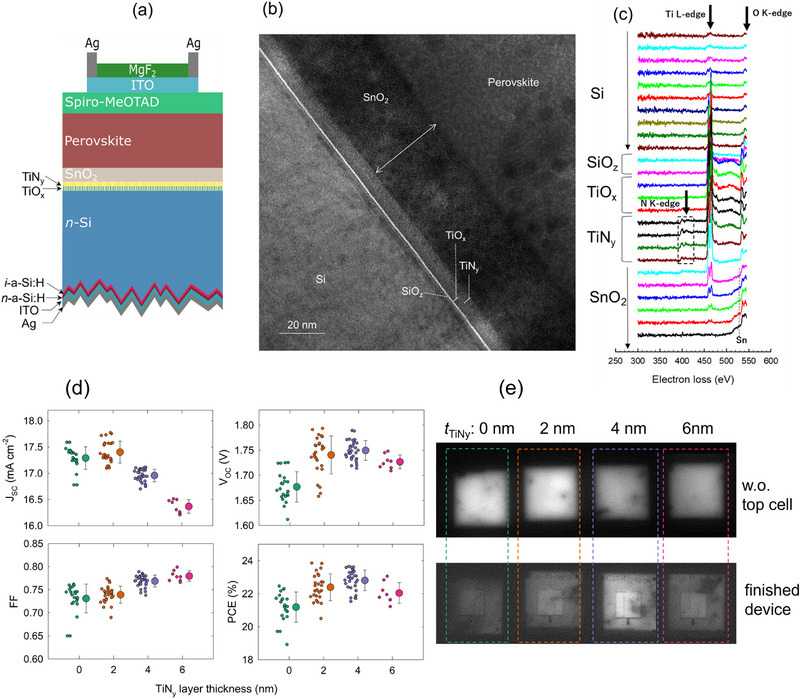
ALD‐TiN_y_ capping layer improves tandem solar cell performance. a) Schematic device structure of the TiO_x_ tandem with a TiN_y_ capping layer. b) Cross‐sectional BF‐TEM image taken at the top/bottom cell interface of the TiO_x_ tandem with a TiN_y_ capping layer. c) EELS spectra measured at different positions near the top/bottom cell interface. Signals due to Ti L edge, O K‐edge, N K‐edge are shown. A broad signal appearing at ∼530 eV originates from Sn. Each line was averaged over the three EELS lines. d) Solar cell parameters of TiO_x_ tandem solar cells with different TiN_y_ thicknesses. Two to seven cells fabricated under identical conditions were measured. Each cell was tested twice under both forward and backward scans. e) PL image of the TiO_x_ tandem with different TiN_y_ capping layer thicknesses before top cell deposition (upper) and after finishing the whole deposition processes (lower).

**Figure** **4**a,b shows the schematic diagram of the TiO_x_ tandem with a TiN_y_ capping layer and the corresponding cross‐sectional BF‐TEM image taken at the top/bottom interface, respectively. The TEM image is similar to that in Figure 2a except for the additional TiN_y_ layer between the TiO_x_ and SnO_2_ layers. Although their phase boundary is not clearly visible, dashed lines are marked for the TiO_x_ (3.6 nm) and TiN_y_ (4.3 nm) layers, separating them from their slightly different image contrast. This image contrast originates from the presence of the crystalline phase in the TiN_y_ layer, which can be identified in the high‐resolution TEM included in Figure S3 (Supporting Information). To investigate the elemental depth profile, we applied an electron energy loss spectroscopy (EELS) measurement instead of EDX due to the difficulty in separating the overlapping x‐ray signals of N and Ti atoms in the EDX measurement. Figure 4c shows the EELS profiles at different depths (∼0.75nm step) in the whole region of the Si/TiO_x_/TiN_y_/SnO_2_ stack. A weak but clear N K‐edge signal is identified in the TiN_y_ layer. In contrast, the much clearer peak due to O K‐edge appears in this layer, indicating that a significant incorporation of O atoms occurs. This is caused by the post‐deposition processes that include annealing in a low vacuum, SnO_2_ spin‐coating, and the subsequent hotplate annealing in air. Despite the O incorporation, we name this layer TiN_y_ for simplicity, as the material was deposited without any oxygen‐containing precursor during the ALD process.

Figure 4d shows the solar cell parameters of the TiO_x_ tandem as a function of the thickness of the TiN_y_ layer (*t*_TiNy_). An adverse effect due to the TiN_y_ layer is seen in J_SC_ when *t*_TiNy_ is greater than 2 nm. We confirmed the presence of broadband light absorption in the 6‐nm‐thick TiN_y_ layer (Figure S4, Supporting Information), accounting for the large J_SC_ drop of 1 mA cm^−2^. However, when *t*_TiNy_ ∼ 4 nm, almost no optical absorption is identified similar to the TiO_x_ layer. This implies that the abrupt increase in optical absorption for *t*_TiNy_ ∼ 6 nm can be associated with the increased nucleation density and the following crystalline growth of TiN_y_. We further confirmed that *t*_TiNy_ ∼ 4 nm is a critical thickness below which no J_sc_ loss is identified in our optimized tandem solar cells (Table S2 and Figure S5, Supporting Information). On the other hand, V_OC_ and FF show a clear increase with increasing *t*_TiNy_ up to 4 nm. As a result, an overall PCE gain of ≈1% absolute is obtained by applying the ∼4 nm‐thick TiN_y_ capping layer. It should be added that the insertion of the TiN_y_ layer at the TiO_x_/SnO_2_ interface does not alter the electrical contact properties, as confirmed by the dark J‐V measurements on the test structure shown in Figure 3a (Figure S6, Supporting Information). Furthermore, it does not alter the Si single‐junction solar cell performance either (Figure S7, Supporting Information).

To gain insight into the role of the TiN_y_ layer in the TiO_x_ tandem, the impact of *t*_TiNy_ on the Si bottom cell performance was investigated by photoluminescence (PL) imaging. Here, carrier injection into the Si bottom cell took place in the tandem structure by illuminating the Si with an infrared laser (850 nm) through the perovskite top cell. Figure 4e shows the PL images of the TiO_x_ tandem with various *t*_TiNy_ before the perovskite *n‐i‐p* top cell deposition and after completing the device fabrication including front ITO/Ag‐grid sputtering. Here, the PL intensity reflects the magnitude of radiative recombination of photogenerated electron‐hole pairs in Si bottom cell, and it decreases when the rate of the non‐radiative recombination via surface defects increases. It is evident from Figure 4e that the finished device without a TiN_y_ layer exhibits the darkest PL image, indicating that the surface recombination rate of photogenerated carriers increases in the Si bottom cell after depositing the top cell component layers. We confirmed that the PL darkening does not occur after SnO_2_ deposition on the TiO_x_ layer, but after depositing the perovskite layer. SCAPS‐1D simulation indicates that the hole inversion near the *n*‐Si/TiO_x_ interface is not affected by the presence of the perovskite layer over the SnO_2_ ETL (Figure S8, Supporting Information). Thus, the band bending lowering in the *n*‐Si by the perovskite overlayer is unlikely. We further confirmed that the PL darkening does not occur in the reference tandems. Based on these facts, we hypothesize that the migrating ionic species of the perovskite (such as Pb, I, and Br) to the TiO_x_ through the porous SnO_2_, which is evidenced by the EDX measurements (Figure 2c), causes a chemical reaction with the TiO_x_ layer and/or the electrostatic influence on the charge distribution near the *n*‐Si/TiO_x_ interface. Thus, the TiN_y_ layer is likely to work as a barrier to prevent such species from contacting the TiO_x_ layer. Further investigation is necessary to elucidate the detailed mechanism.

**Figure 5:**
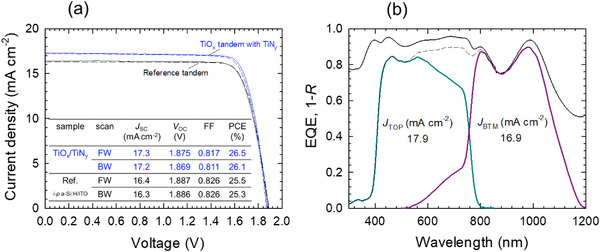
The best performing tandem solar cells. a) J‐V curves of a best‐performing TiO_x_ tandem with a TiN_y_ layer between the TiO_x_ and SnO_2_ layers (blue lines). The J‐V curves of a best‐performing reference tandem (black lines) after the process optimization, which are superior to those in Figure 1e, are also shown for comparison. Solid and dashed lines represent the forward (FW) and backward (BW) scans, respectively. b) EQE spectra of the top and bottom cells of the corresponding TiO_x_ tandem. The summed EQE spectrum is shown in dashed line. Absorption spectrum (1‐ reflectance (*R*)) measured by spectrometer is included (solid black line).

Along with fine‐tuning the TiO_x_ and TiN_y_ deposition processes, we have optimized the tandem cell fabrication process. Notably, changing the Ag‐grid formation method from DC sputtering to thermal evaporation results in improved performance (particularly V_OC_ and FF) and reproducibility, likely due to the mitigation of sputter‐induced damage. This leads to a performance improvement in both TiO_x_ and reference tandems. The J‐V curves and the external quantum efficiency (EQE) spectra of the best‐performing TiO_x_ tandem with a TiN_y_ capping layer are shown in **Figure** **5**a (blue lines) and Figure 5b, respectively. The PCE of this device under forward scan is 26.5% (J_SC_ = 17.3 mA cm^−2^, V_OC_ = 1.875 V, FF = 0.817). A sister device fabricated in an earlier batch under the same condition showed a PCE of 24.0% (J_SC_ = 16.8 mA cm^−2^, V_OC_ = 1.778 V, FF = 0.809) in in‐house measurements, and a PCE of 24.80% (J_SC_ = 17.4 mA cm^−2^, V_OC_ = 1.784 V, FF = 0.798) with a steady‐state PCE of 24.82% via independently‐confirmed measurements (Figure S9, Supporting Information). In Figure 5a, the TiO_x_ tandem exhibits a higher PCE (26.5%) in comparison with the improved reference tandem (25.5%) due to its higher J_SC_. The higher J_SC_ of the TiO_x_ tandem comes from the batch‐to‐batch variation in the thickness of the top cell component layers (mainly the HTL thickness). On the other hand, the V_OC_ achieved in the TiO_x_ tandem is 1.875 V which is comparable to that in the reference tandem (1.887 V). This manifests that the TiO_x_/TiN_y_ bilayer provides comparable Si passivation, hole extraction, and RJ functions to those achieved with the a‐Si:H passivating contact layers and an ITO interlayer.

Previously, it has been reported that the ITO interlayer can be replaced by an *n*‐type hydrogenated nanocrystalline Si (nc‐Si:H) layer.^[^ ^29^ ^]^ Replacing ITO with nc‐Si:H provides a PCE of 24.4% in the same device design but without applying the latest process optimization such as Ag‐grid formation by evaporation (Figure S10, Supporting Information). The nc‐Si:H also offers an indium‐free interconnection although it still requires the use of an SHJ bottom cell architecture.

Although recent tandem cell development focuses more on an inverted *p‐i‐n* configuration, we discuss our tandem cell results in comparison with the state‐of‐the‐art tandem devices with an *n‐i‐p* configuration. Among the *n‐i‐p* tandem solar cells reported so far,^[^ ^38^, ^39^, ^40^, ^41^, ^42^ ^]^ our device performance is lower by ∼1–3% absolute. This mainly stems from the following reasons: First, our perovskite top cell was made without any interface passivation. The performance of our perovskite single‐junction solar cell using the same recipe as for the top cell is about PCE ∼ 17–18% (Figure S2, Supporting Information). The 27%‐efficient tandem device in the literature has a perovskite top cell with higher performance (PCE > 20% single‐junction efficiency).^[^ ^38^ ^]^ Second, the band gap of the perovskite absorber (1.63 eV) is not optimized for tandem solar cells (∼1.7 eV). Third, the performance of our TiO_x_ tandem is limited by the low J_SC_ (∼17 mA cm^−2^) due to the larger optical reflection (Figure 5b), compared to the J_SC_ (∼20 mA cm^−2^) of the state‐of‐the‐art tandem cells.^[^ ^38^, ^40^, ^41^, ^42^ ^]^ The replacement of thick spiro‐MeOTAD (∼200 nm) with similar derivatives such as evaporated 2,2′,7,7′‐tetra(N,N‐di‐p‐tolyl)amino‐9,9‐spirobifluorene (spiro‐TTB) with much lower thickness (∼25 nm) would improve the J_SC_ and PCE of our devices. In addition, the introduction of a nanoscale texture that allows uniform top cell formation by the solution process should also increase J_SC_.^[^ ^6^, ^43^, ^44^ ^]^

The aim of this study is to extend the developed TiO_x_ technology to commercial homojunction bottom cells such as PERC and TOPCon. In both cell architectures, the TiO_x_ proposed here can interconnect the hole‐collecting side of the Si bottom cell (e.g., *p*‐Si absorber of PERC and boron‐doped Si in TOPCon) with the perovskite top cell deposited in an *n‐i‐p* sequence. In the case of TOPCon based on *n*‐Si absorber, TiO_x_ can be used as an emitter without creating a boron doped emitter. This offers the opportunity to combine low‐cost industrial Si solar cells with perovskite solar cells via the TiO_x_ transparent passivating contact, without the need for indium‐oxide‐based interlayers. This has crucial implications for the design of terawatt‐scale sustainable tandem devices.

## 3 Conclusion

We have demonstrated that an ALD‐TiO_x_ nanolayer enables direct interconnection between an *n‐i‐p* perovskite top cell and an *n*‐Si wafer, achieving a PCE of 22.4% despite the significantly simplified tandem device structure. We show that the TiO_x_ layers are multifunctional, fulfilling the requirements in Si surface passivation, hole extraction from Si, and RJ at the top/bottom cell interface. The introduction of a partially‐crystallized ALD‐TiN_y_ capping layer on top of the TiO_x_ layer improves the *n*‐Si/TiO_x_ interface passivation, achieving a PCE up to 26.5% which is comparable with the performance of the tandem cells that use a conventional SHJ bottom cell with an ITO interlayer (PCE = 25.5%). This result offers the potential for lowering the manufacturing cost of perovskite/Si tandem solar cells as well as the application of industrial Si solar cells (such as PERC and TOPCon) in tandem solar cell designs.

## 4 Experimental Section

### ALD Processes for TiO_x_ and TiN_y_ Layers

TiO_x_ layers were deposited by a custom‐made ALD chamber equipped with an inductively‐coupled plasma source and a load‐lock chamber (Eiko Corporation). TiO_x_ layers were grown by thermal ALD at a substrate heater temperature of 300 °C using titanium tetraisopropoxide (TTIP) and H_2_O vapor as a Ti precursor and an oxidant, respectively. TTIP source temperature was kept at 60 °C in a constant temperature bath and it was delivered to the chamber by an Ar bubbler system. TTIP dose time was 1.2 s. The H_2_O dose was controlled by the valve opening time (1.2 s) and the number of H_2_O pulses (3 times) in an ALD cycle. By repeating ALD cycle for 128 times, ∼5 nm‐thick TiO_x_ layer was deposited. Then, the TiO_x_ layer underwent a hydrogen plasma treatment (HPT) in the same ALD chamber under a pressure of 10 Pa, a H_2_ flow rate of 100 SCCM, and an rf plasma power of 600 W.

As mentioned in the main text, the TiN_y_ layers were deposited in a different ALD system (FlexAL, Oxford Instruments) from the above‐mentioned ALD system for the TiO_x_ layer deposition due to the lack of access to the NH_3_ gas. The TiN_y_ layers were grown at a substrate heater temperature of 300 °C using TDMAT and NH_3_ as a Ti precursor and a reactant gas, respectively. In similar to TTIP, TDMAT source temperature was kept at 60 °C and it was delivered to the chamber by an Ar bubbler system. The dose times for TDMAT and NH_3_ were 1.2 and 6 s, respectively. By repeating ALD cycle for 50 times, ∼4 nm‐thick TiN_y_ layer was deposited.

### Solar Cell Fabrication

Planar monocrystalline Si wafers (phosphorous‐doped *n*‐type float zone Si, 2–3 Ωcm, (100) orientation, 280 µm thick) were used in this study. The front‐planar and rear‐textured structure was prepared by the following process step. First, a SiN_x_ layer was deposited on the single side of the Si wafer by plasma‐enhanced chemical vapor deposition (PECVD). This SiN_x_ served as a protection layer during the following etching process. Then, Si wafers were subjected to wet‐chemical etching in a KOH‐based solution (Hayashi Pure Chemical, Pure Etch TP101) to form random pyramidal textures on the uncoated side. Finally, the SiN_x_ film was stripped by immersing the wafers in a diluted HF solution.

The wafers were cleaned using H_2_SO_4_, H_2_O_2_, HCl, and HF.^[^ ^45^ ^]^ Then, an intrinsic and phosphorous‐doped (*n*‐type) a‐Si:H layer stack was deposited as an electron‐selective passivating contact on the textured side of the Si wafers by means of PECVD operated at 13.56 MHz using SiH_4_, PH_3_, and H_2_ as source gases.^[^ ^45^ ^]^ After HF‐dip, TiO_x_ layer was deposited by ALD as a hole‐selective passivating contact on the planar side of the Si wafers. After HPT, some of the samples were capped with a TiN_y_ layer. As a reference, a standard SHJ solar cell was prepared by depositing an intrinsic and boron‐doped (*p*‐type) a‐Si:H layers, instead of the TiO_x_ layer. A 5‐nm‐thick ITO layer was deposited on a *p*‐a‐Si:H layer as an interlayer through a shadow mask whose aperture area was designed to have a slightly larger area than the front‐side ITO layer. As for the textured rear side, ITO (70 nm) and Ag (500 nm) layers were formed by magnetron sputtering for the entire area of the samples without patterning. Then, these Si bottom cells were thermally annealed at 180 °C in a low vacuum oven (∼10 Pa of air).

Prior to spin‐coting of the SnO_2_ layer, the front surface of bottom cells was treated using two different methods. For the TiO_x_ tandem, the surface of the TiO_x_ or TiN_y_ layer was exposed to the same HPT in the ALD system as used for treating the TiO_x_ surface. However, the plasma power was 300 W. For the reference tandem, the front surface of the SHJ bottom cells was treated with UV‐ozone (SAMCO, UV‐1). UV ozone was not used for the TiO_x_ tandem to avoid UV‐induced passivation degradation.^[^ ^19^ ^]^ For SnO_2_ spin coating, an SnO_2_ nanoparticle colloidal solution (Taki Chemical) was diluted with deionized water with a volume ratio of 1:0.5, and then 300 µL of the solution was spin‐coated (2000 rpm for 30 s) followed by hotplate annealing at 100 °C on a hot plate in dry air for 1 h. This results in a ∼40‐nm‐thick SnO_2_ layer. For perovskite layer, Rb_0.05_(FA_0.83_MA_0.17_)_0.95_Pb(I_0.83_Br_0.17_)_3_ perovskite^[^ ^29^, ^46^ ^]^ was prepared by dissolving 1.4 m PbI_2_, 0.25 m PbBr_2_, 0.09 m RbI, 0.25 m methylammonium bromide (MABr), and 1.26 m formamidinium iodide (FAI) in a 4:1 (V:V) mixture of dimethylformamide (DMF) and dimethylsulfoxide (DMSO). A 10% molar excess of methylammonium chloride (MACl) was added to the solution. The solution was mixed overnight at room temperature and used the next day. An amount of 60 µL of the solution was pipetted onto the substrate and spin‐coated in a two‐step program: 1300 rpm for 5 s with 200 rpm s^−1^ ramp and then 5000 rpm for 30 s with 2000 rpm s^−1^ ramp. Ten seconds before the end of the second step, 300 µL of anhydrous anisole was pipetted onto the rotating substrate. The substrates were then annealed at 110 °C for 15 min. The HTL was prepared by dissolving 72 mg of spiro‐MeOTAD (Nippon Fine Chemical) in 1 mL of chlorobenzene. The spiro‐MeOTAD solution was doped by adding 17.5 µL of Li‐TFSI (520 mg mL^−1^ in acetonitrile) and 28.5 µL of 4‐tertbutylpyridine to the spiro‐MeOTAD solution. The solution was shaken at room temperature for 3 h prior to use. An amount of 50 µL of the doped spiro‐MeOTAD solution was pipetted onto the substrate and spun at 4000 rpm for 30 s. Devices were then placed in dry air (<1% relative humidity) over night. Next, a ∼100‐nm‐thick ITO (In_2_O_3_/SnO_2_ 96.5/3.5 wt.% in target) was sputtered through a shadow mask in an Ar−O_2_ (O_2_: 0.5%) gas mixture using a long‐throw magnetron rf‐sputtering system (CANON‐ANELVA, E‐400S). Here, no buffer layer was used between HTL and ITO layer. To mitigate the sputter‐induced damage, a long target‐substate distance of 250 mm was used. The Ag finger and busbar were formed either in the same sputtering system or in a different thermal evaporation system. The Ag was capped with an ITO (20 nm) thin layer to protect underlying Ag from corrosion during storage. The resulting device area was ∼1 cm^2^. The tandem devices were coated with a ∼100 nm‐thick MgF_2_ AR layer using a vacuum evaporator (Moorfield, MiniLab 090). Finally, the devices were then placed in a N_2_ ‐purged oven at 50 °C for 1 h.

### Characterization

The effective minority carrier lifetime and the implied V_OC_ (iV_OC_) of the Si solar cell precursors (before metallization) were measured with a transient photoconductance measurement setup (Sinton Instruments, WCT‐120). For the finished tandem cells, the J‐V characteristics were evaluated at 25 °C using a dual‐light source class AAA sun simulator (Wacom, WXS‐50S‐L2 or Sanei‐Denki, XHS‐80S1) with an air mass 1.5 global (AM1.5 g) irradiance spectrum at 100 mW cm^−2^. For perovskite single‐junction solar cells, a Xe lamp sun simulator (AM1.5 g, 100 mW cm^−2^) was used (Bunkou‐Keiki, OtentoSun). The light intensity of sun simulator was calibrated using calibrated reference Si solar cells (the one used for perovskite single‐junction cell has an IR cut filter). The illumination area of 1.0 cm^2^ was designated by using a black shadow mask whose aperture area was designed to be slightly smaller than the area of the front ITO electrode. The J‐V parameters of solar cells were recorded using a source meter (Keithley 2400 or 2401) in the voltage range from −0.1 to 1.9 V without any voltage soaking or light soaking. The scan rate used was 0.125 V s^−1^. All devices were tested in air at room temperature (25 °C). The EQE spectrum was measured in alternating current (AC) mode using a monochromatic light (Bunkou‐Keiki, CEP‐25BXS). The step size was 10 nm. When measuring perovskite top cells, light bias (λ>750 nm) was applied via a blue‐cut filter to saturate the bottom‐cell current; when measuring Si bottom cells, the tandem devices were light‐biased via a red‐cut filter (λ<450 nm) to saturate the top‐cell current. In addition to the light bias, a forward bias voltage (V_bias_), which is equivalent to the V_OC_ of the saturated (untested) subcell, was applied (for top cell: V_bias_ = 0.7 V, for bottom cell: V_bias_ = 1.2 V). When measuring bottom cell EQE, a small signal arising from the perovskite top cell (EQE<0.05) appears for wavelength of 400–800 nm, particularly for the TiO_x_ tandems. Such an artifact was corrected after the measurements.

Dark J‐V test structures were fabricated on planar 280‐µm‐thick 3 Ωcm *p*‐type FZ Si wafers. To form an ohmic contact at the rear side of the wafer, a 25‐nm‐thick *p*‐a‐Si:H layer (same doping type with the wafer) and a sputtered Ag (600 nm) were deposited. The TiO_x_ and SnO_2_ layers stack under investigation was deposited on the front side of the Si wafer, and then capped with six pads of evaporated Al layers (500 nm) with an area of 0.25 cm^2^. Two‐terminal J‐V measurements were performed using a Keithley 6430 source meter.

The PL measurement was carried out in an ITES PVX1000 + POPLI‐Λ system. The samples were homogeneously illuminated by an excitation laser with a wavelength of 850 nm through a beam expander and a PL signal was recorded in a Si‐CCD camera with a cut‐on wavelength of 990 nm.

TEM, EDX, and EELS measurements were carried out at JFE Techno‐Research Corporation, Japan. Cross‐sections of the samples were observed by HAADF‐STEM or bright‐field TEM imaging combined with EDX spectroscopy at an acceleration voltage of 80 kV (JEOL JEM‐ARM200F). To avoid the electron‐beam induced degradation of the samples, the EDX scanning was carried out under the minimum irradiation condition.

## Conflict of Interest

The authors declare no conflict of interest.

## Author Contributions

T.M. supervised the project, designed the experiments, performed the ALD processes, thin‐film coatings, and measurements at AIST, and analyzed the experimental data. C.M. and M.A. performed perovskite single‐junction solar cell development and characterizations. H.S. constructed the baseline processes and characterizations for crystalline Si solar cells at AIST. J.M. and R.S.B. carried out device simulations. T.M. wrote the manuscript. The manuscript was corrected by R.S.B., C.M., A.M., and H.S. All authors discussed the results and provided comments on the manuscript.

## Supporting information



Supporting Information

Supplemental Table

## Data Availability

The data that support the findings of this study are available from the corresponding author upon reasonable request.
