# Supplementary material for: Monolithic Perovskite/Silicon Tandem Solar Cells Enabled by Multifunctional TiOx Interconnects
Source: Small. 2025 Apr 27;21(24):2500969. doi: 10.1002/smll.202500969 (PMC12177845; doi:10.1002/smll.202500969)
Supplement: Supplementary file 1 — Supporting Information [file SMLL-21-2500969-s001.docx]

**Supporting Information**

**Monolithic Perovskite/Silicon Tandem Solar Cells Enabled by Multifunctional TiO_x_ Interconnects**

Takuya Matsui,^*1^ Calum McDonald,^1^ Abduheber Mirzehmet,^1^ James McQueen,^2^ Ruy Sebastian Bonilla^2^, Hitoshi Sai^1^

^1^Renewable Energy Advanced Research Center, National Institute of Advanced Industrial Science and Technology (AIST), 1-1-1 Umezono, Tsukuba, Ibaraki, 305-8568, Japan

^2^Department of Materials, University of Oxford, 16 Parks Rd, Oxford, OX1 3PH, United Kingdom

* e-mail: t-matsui@aist.go.jp


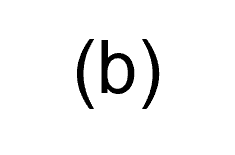

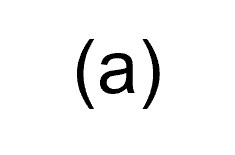

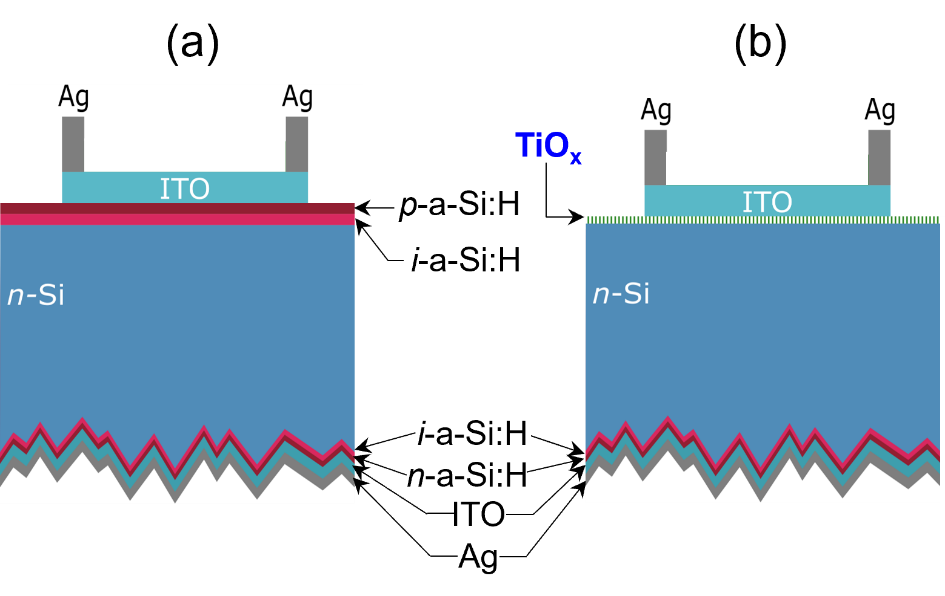

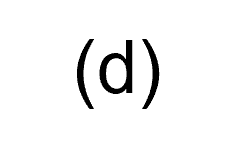

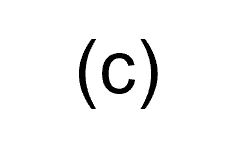

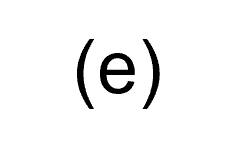

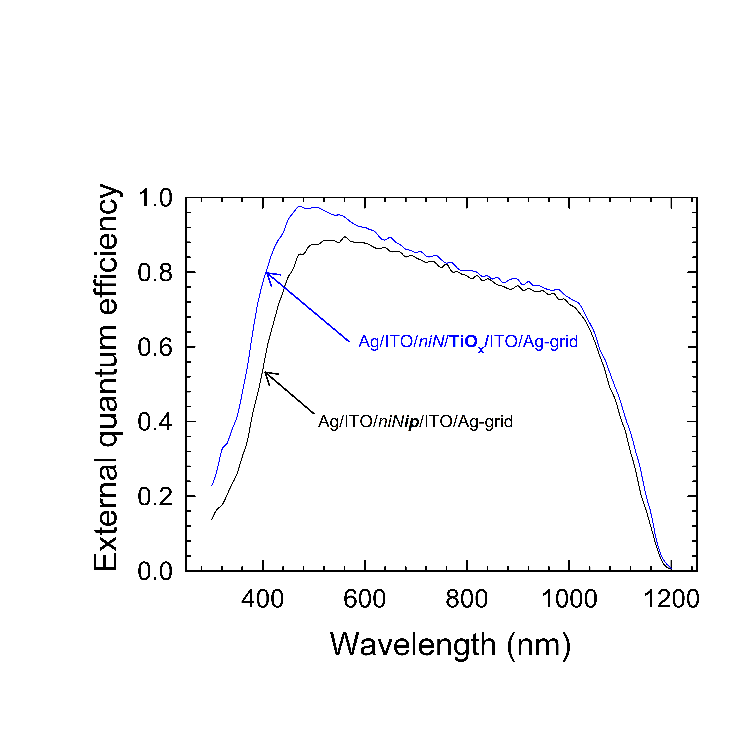

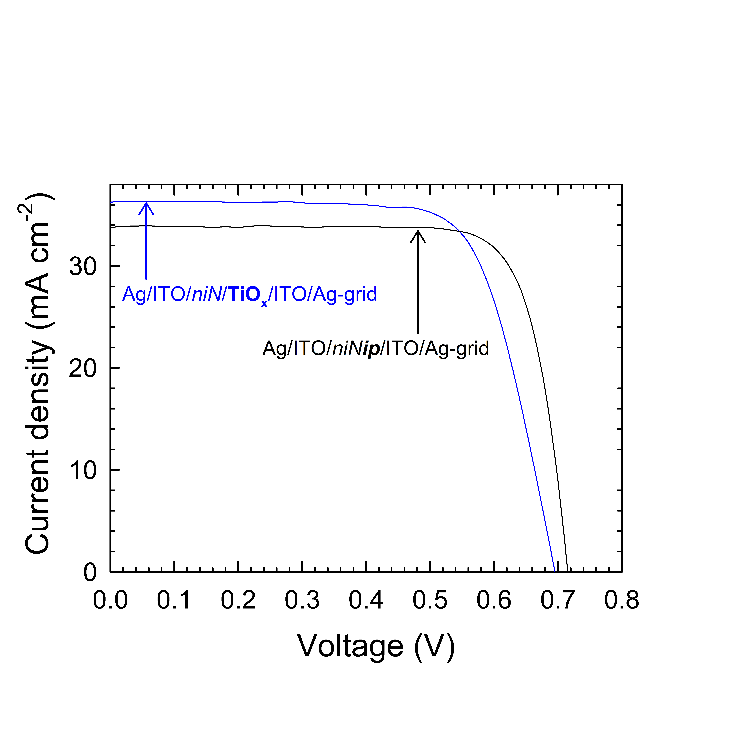

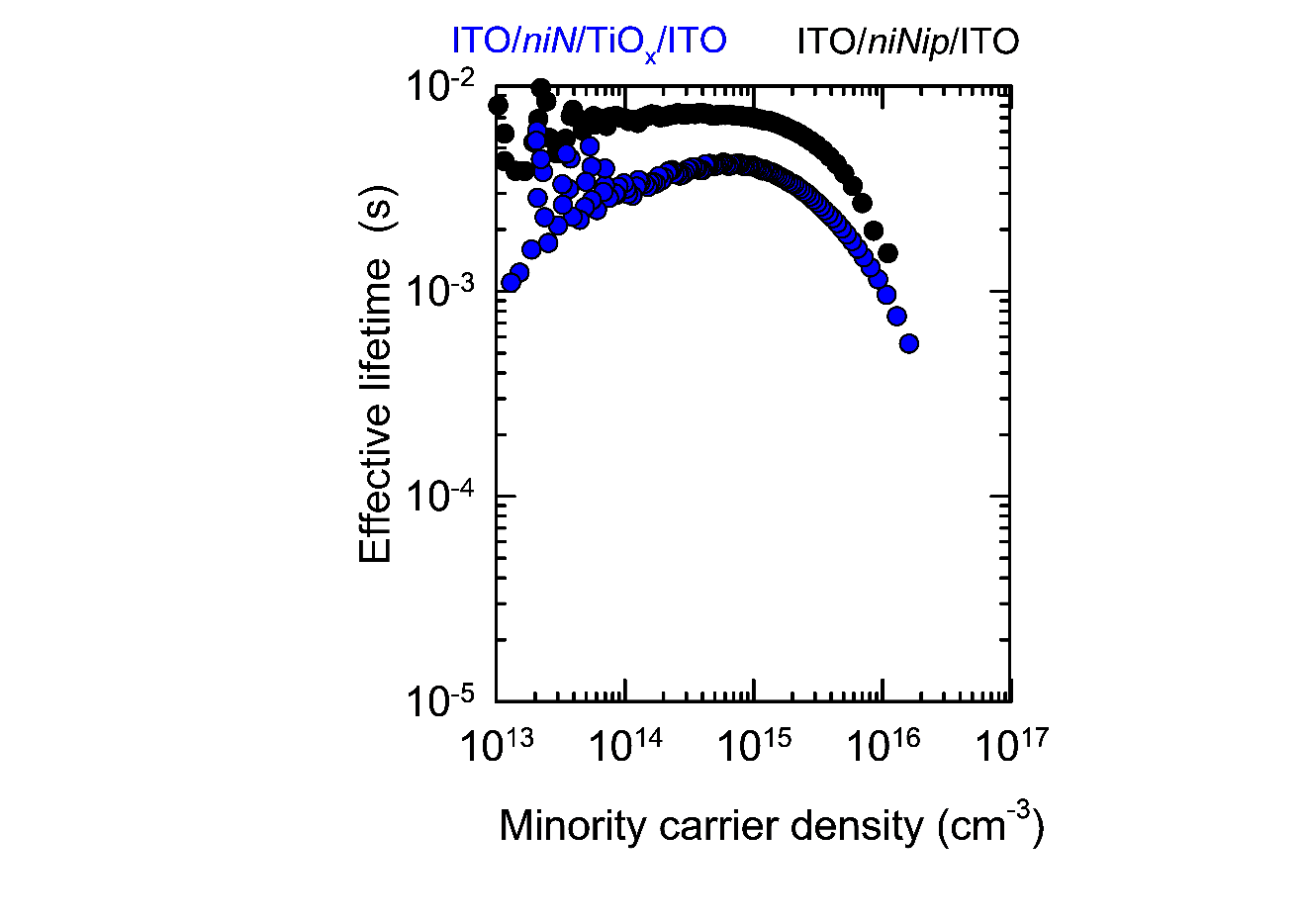

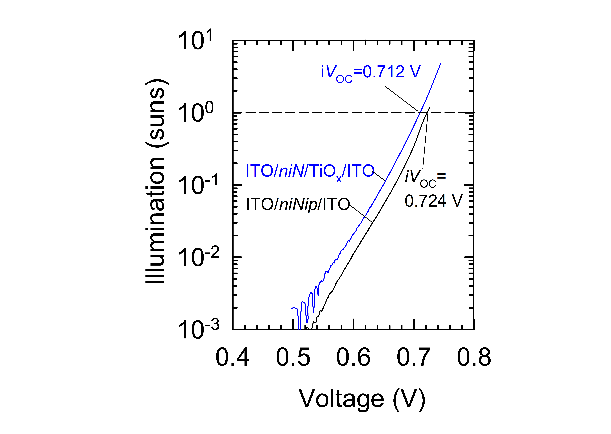


**Figure S1**. (a, b) Schematic illustrations of Si single-junction solar cells whose J-V curves are shown in Figure 1c of the main text ((a) standard SHJ solar cell and (b) TiO_x_-Si single-junction solar cell). (c) Injection dependent minority carrier lifetime curves for devices shown in (a) and (b) (before Ag metallization) measured by transient photoconductance. Inset shows the suns-V_OC_ curves. (d) J-V curves and (e) EQE spectra of the TiO_x_-Si cell and the standard SHJ cell.

**Figure S1**. (a, b) Schematic illustrations of Si single-junction solar cells whose J-V curves are shown in Figure 1c of the main text ((a) standard SHJ solar cell and (b) TiO_x_-Si single-junction solar cell). (c) Injection dependent minority carrier lifetime curves for devices shown in (a) and (b) (before Ag metallization) measured by transient photoconductance. Inset shows the suns-V_OC_ curves. (d) J-V curves and (e) EQE spectra of the TiO_x_-Si cell and the standard SHJ cell.

**Figure S2**. (a) Schematic illustration of perovskite single-junction solar cell. (b) J-V curves and (c) EQE spectrum of the typical device. In (c), band gap of absorber layer was determined from the first derivative of the EQE spectrum.^[1]^


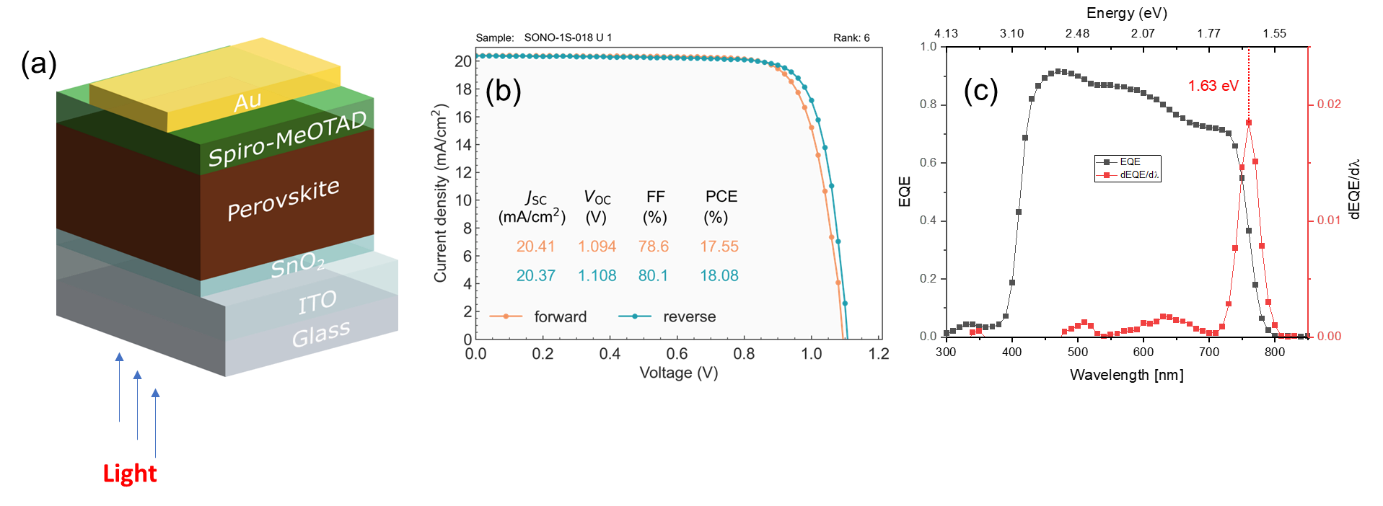


**Table S1**. Material parameters assumed in the SCAPS-1D simulations.

| Material parameters | *p*-Si | *n*-Si | TiO_x_ | SnO_2_ | perovskite |
| --- | --- | --- | --- | --- | --- |
| Bandgap (eV) | 1.12 | 1.12 | 3.5 | 3.6 ^[3]^ | 1.63 |
| Electron affinity (eV) | 4.05 | 4.05 | 4.0 ^[2]^ | 4.5 ^[3]^ | 4.2 ^[6]^ |
| Relative permittivity | 11.9 | 11.9 | 15.0 | 10 ^[3]^ | 24 ^[7]^ |
| Electron concentration (cm^-3^) | - | 1.5×10^15^ | 1.0×10^13^ | 1.0×10^18 [4,5]^ | 1.0×10^9^ |
| Hole concentration (cm^-3^) | 1.5×10^15^ | - | - | - | 1.0×10^9^ |

**Figure S4**. Absorbance (1-Reflectance-Transmittance) spectra of TiO_x_ (5 nm) and TiN_y_ (4 and 6 nm) layers deposited on glass substrates. A spectrum of glass substrate is shown for comparison (black dashed line). A marked optical absorption occurs when the thickness of TiN_y_ layer is greater than 6 nm probably due to the increased crystalline phase in the TiN_y_ layer. Note that the growth of such thin layers on glass could differ from that on Si.


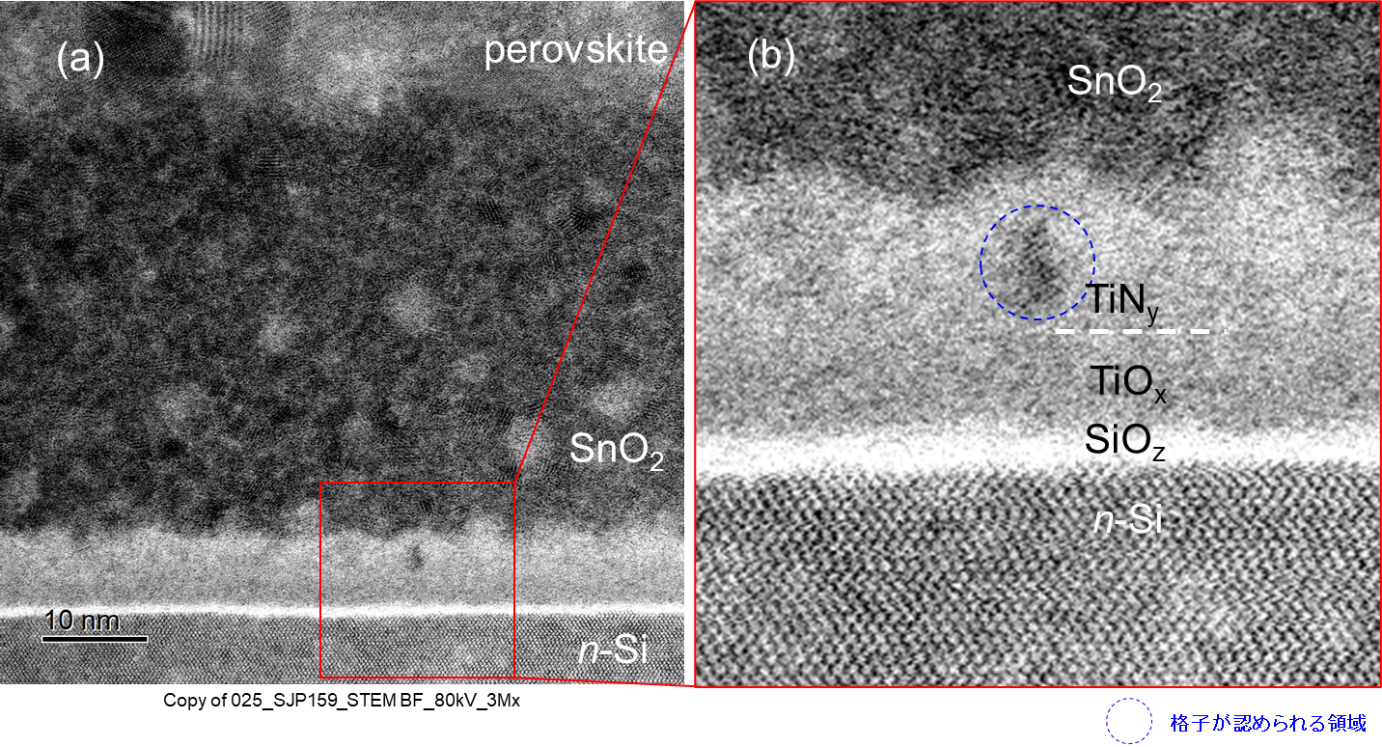


**Figure S3**. (a) Cross-sectional bright-field scanning transmission electron microscopy image and (b) its magnified high-resolution image of a stack of *n*-Si/TiO_x_/TiN_y_/SnO_2_ layers. A fringe pattern due to e-beam diffraction is identified in the TiN_y_ layer (blue dashed circle in (b)), showing that TiN_y_ layer contains crystalline phase. In addtion, surface roughning at the TiN_y_/SnO_2_ interface is observed, indicating that the top of the TiN_y_ layer is crystallized.

**Table S2**. Solar cell parameters of the improved TiO_x_ tandems with various TiN_y_ capping layer thicknesses. These TiO_x_ tandems were fabricated after device optimizations described in the main text, and they exhibit higher performance than the results shown in Figure 4d. It is clarified that the J_SC_ decrease occurs when the thickness of the TiN_y_ layer is greater than 6 nm, in agreement with the measured optical absorbance of the TiN_y_ layers shown in Figure S4.

| TiN_y_ thickness (nm) | Scan direction | J_SC_  (mA cm^-2^) | V_OC_  (V) | FF | PCE  (%) |
| --- | --- | --- | --- | --- | --- |
| 2 | Forward | 16.9 | 1.763 | 0.816 | 24.3 |
|  | Backward | 16.9 | 1.762 | 0.814 | 24.2 |
| 4 | Forward | 17.1 | 1.787 | 0.813 | 24.8 |
|  | Backward | 17.0 | 1.785 | 0.813 | 24.7 |
| 6 | Forward | 16.1 | 1.730 | 0.808 | 22.5 |
|  | Backward | 16.1 | 1.722 | 0.789 | 21.8 |


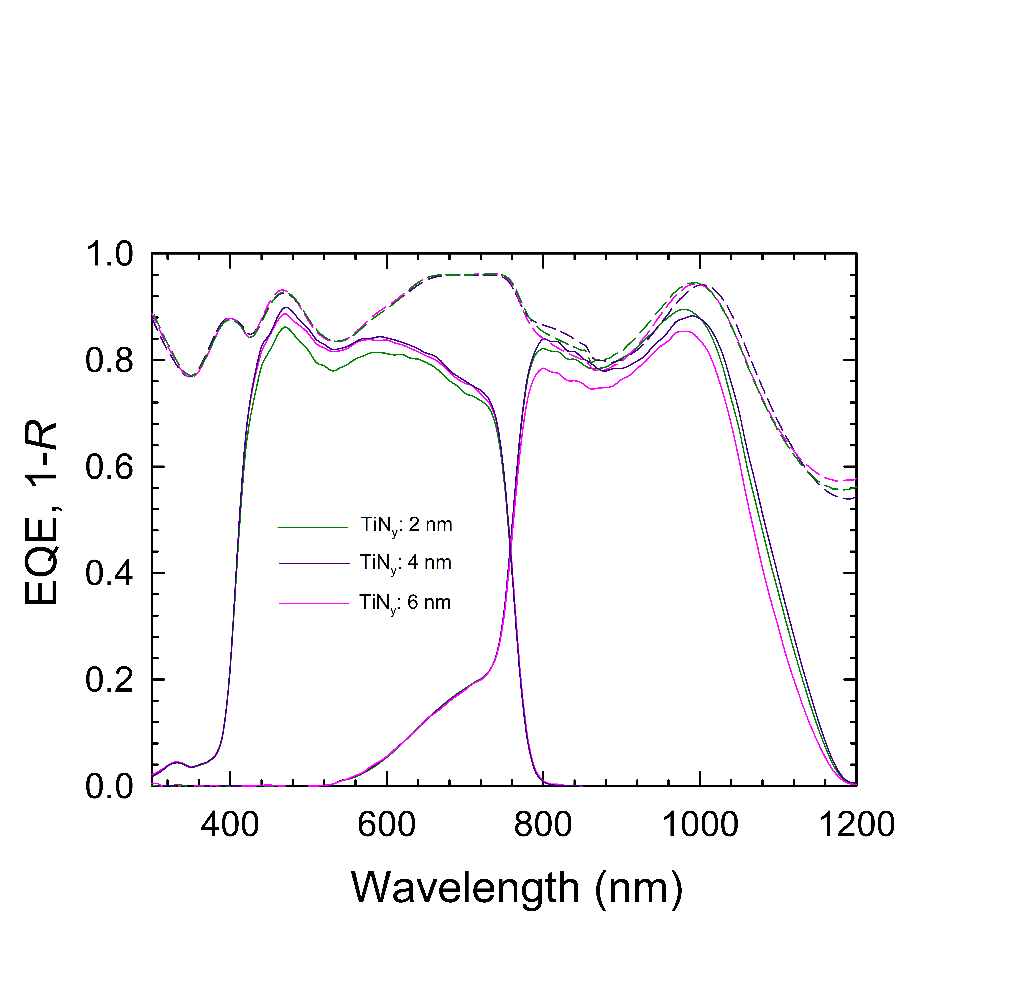


**Figure S5**. EQE spectra of the top and bottom cells of the corresponding TiO_x_ tandems listed in Table S2. Absorption spectra (1- reflectance (*R*)) measured by spectrometer are included (dashed lines). The decrease in the bottom cell response is identified for the device that has a TiN_y_ thickness of 6 nm.

**Figure S7**. (a) J-V curves and (b) EQE spectra of the TiO_x_-Si single-junction solar cells with (red line) and without (blue line) a TiN_y_ capping layer (4 nm) between the TiO_x_ and ITO layers. It is clarified that the 4-nm-thick TiN_y_ capping on the TiO_x_ layer does not essentially alter the Si single-junction solar cell performance.


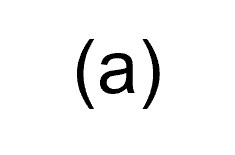

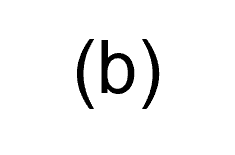


**Figure S6**. (a) Test structure for electrical contact measurement and (b) the dark J-V characteristics of samples with (red lines) and without (blue lines) a 4-nm-thick TiN_x_ layer between the TiO_x_ and SnO_2_ layers. The result indicates that the TiN_y_ capping layer does not alter the electrical contact properties at the top/bottom interface. Note that J-V curves of the sample without the TiN_y_ layer (blue lines) slightly differ from those in Figure 3b probably because a different contact geometry (φ3 mm) was appleid.


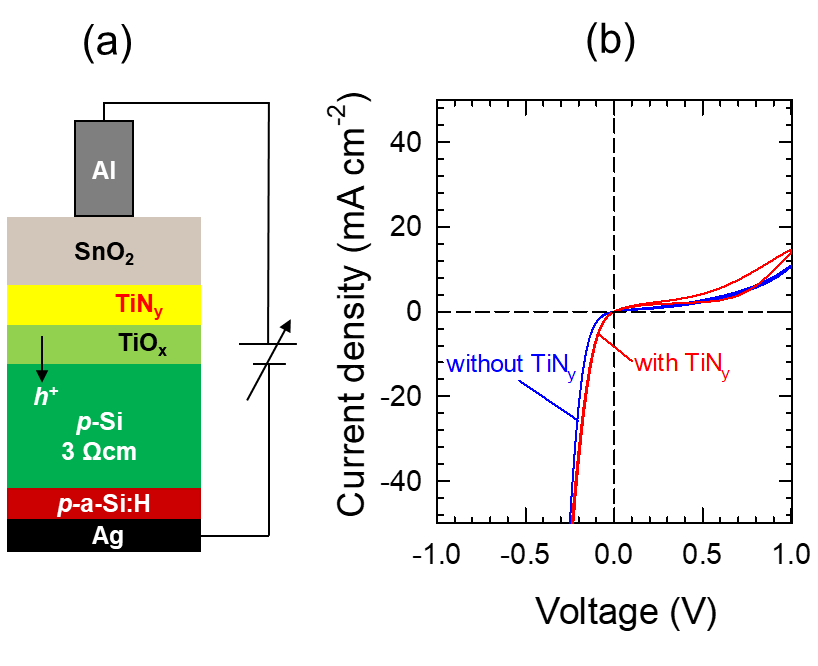


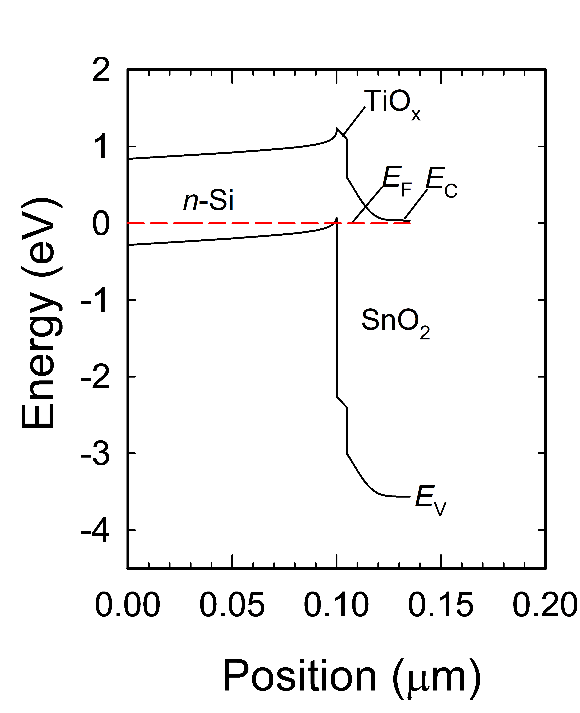

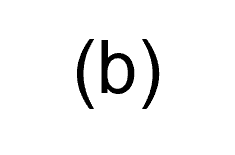

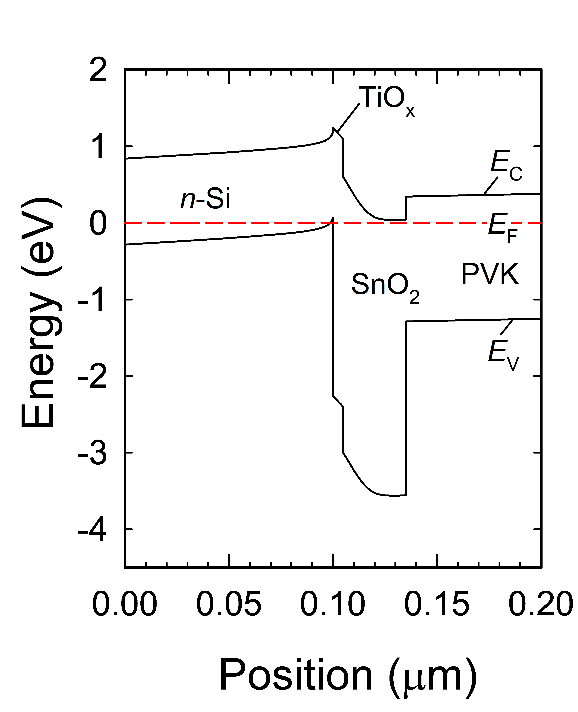

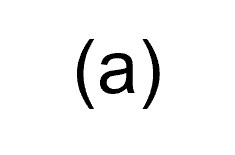


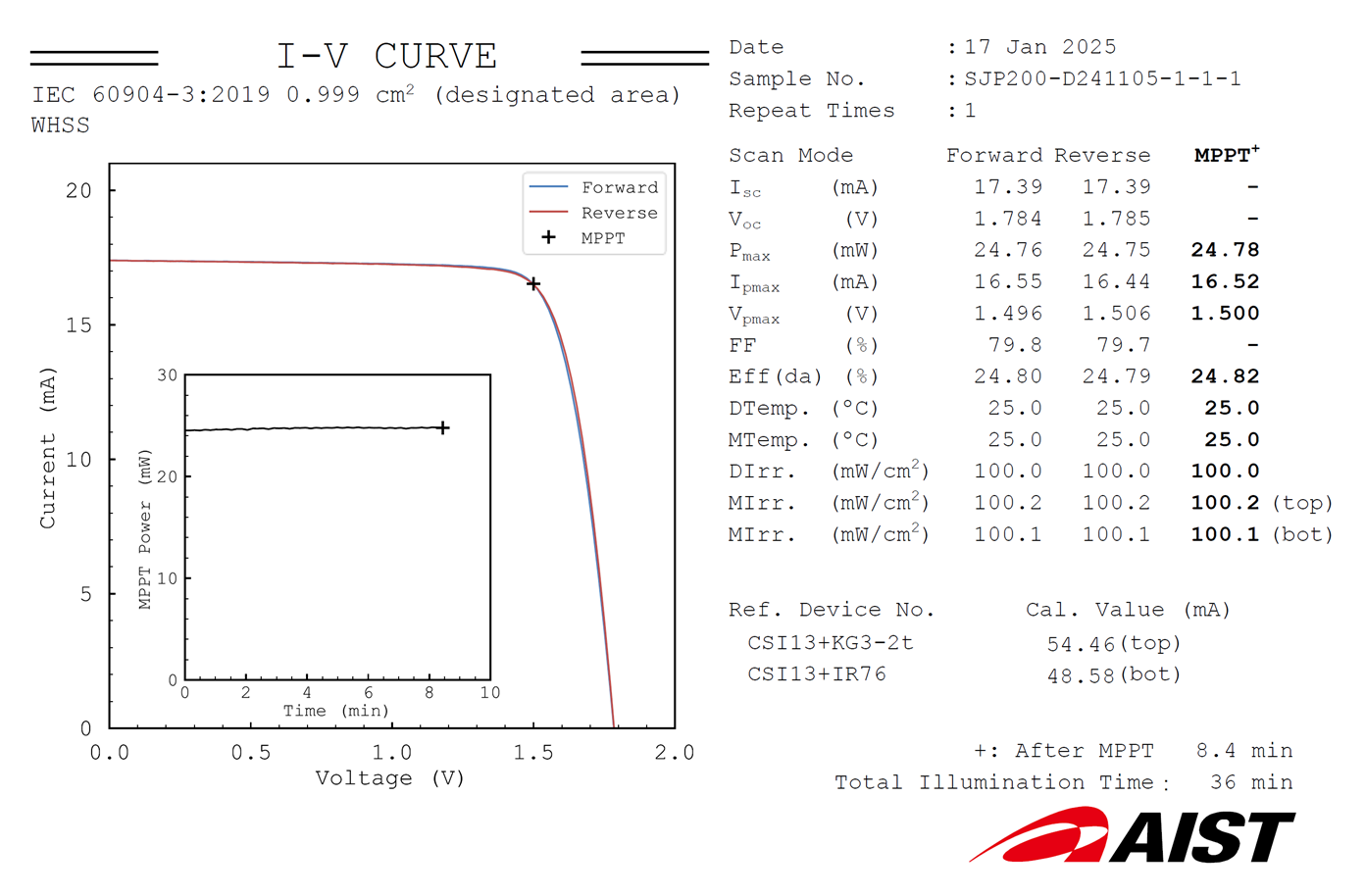


**Figure S9** Illuminated current-voltage characteristics of the TiO_x_ tandem featuring a 4-nm-thick TiN_y_ capping layer measured by the Calibration, Standards and Measurement Team of AIST.

**Figure S8** Band diagrams of tandem device (a) before and (b) after perovskite (PVK) layer formation on top of the SnO_2_ ETL of the top cell, obtained by a SCAPS-1D finite element device simulation. *E*_C_ and *E*_V_ denote energy levels of conduction band and valence band, respectively. *E*_F_ is the Fermi level.

**Figure S10** (a) Schematic illustration of the solar cell featuring *in-situ* grown *n*-type hydrogenated nanocrystalline Si (nc-Si:H) RJ layer between the SHJ bottom cell and the perovskite *n-i-p* top cell. (b) J-V curves of the corresponding tandem cell (solid line: forward scan, dashed line: backward scan). The device was fabricated without applying the latest process optimization such as Ag-grid formation by evaporation.


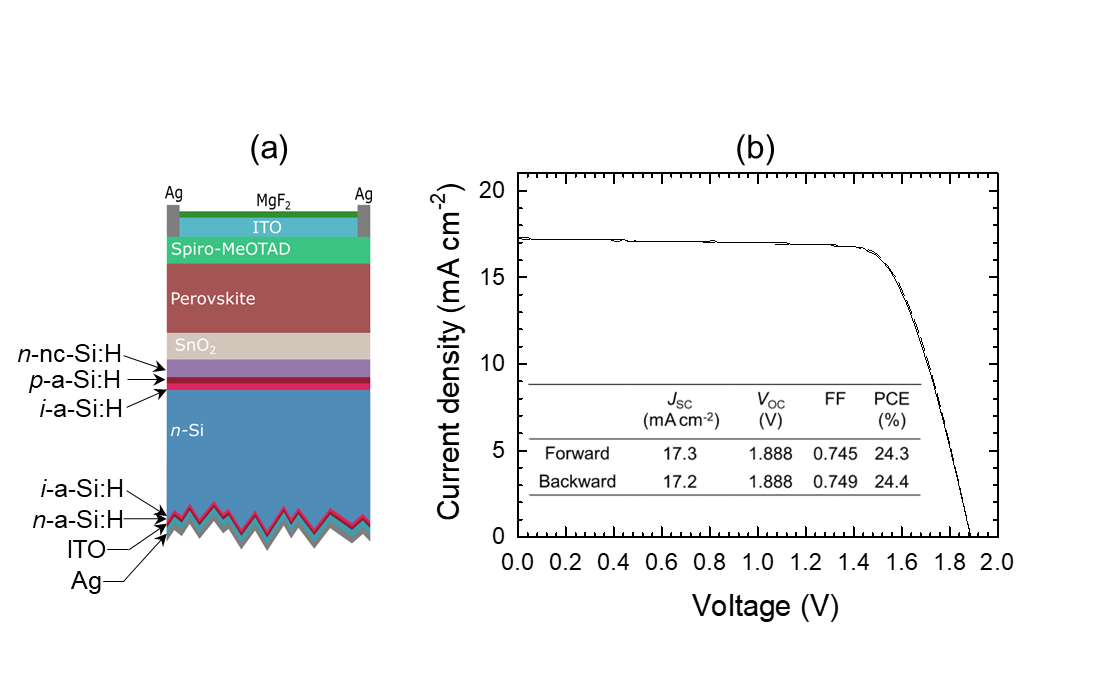


References

1. O. Almora, C. I. Cabrera, J. Garcia-Cerrillo, T. Kirchartz, U. Rau, C. J. Brabec, *Adv. Energy Mater.* **2021**, *11*, 2100022. <https://doi.org/10.1002/aenm.202100022>.
2. F. C. Marques, J. J. Jasieniak, *Appl. Surf. Sci.* **2017**, *422*, 504. <https://doi.org/10.1016/j.apsusc.2017.06.062>.
3. L. Kavan, *J. Solid State Electrochem.* **2024**, *28*, 829. <https://doi.org/10.1007/s10008-023-05770-w>.
4. W. Hui, Y. Yang, Q. Xu, H. Gu, S. Feng, Z. Su, M. Zhang, J. Wang, X. Li, J. Fang, F. Xia, Y. Xia, Y. Chen, X. Gao, W. Huang, *Adv. Mater.* **2020**, *32*, 1906374. <https://doi.org/10.1002/adma.201906374>.
5. M. F. U. Din, S. Sousani, M. Kotlar, S. Ullah, M. Gregor, T. Scepka, Y. Soyka, A. Stepura, A. Shaji, F. Igbari, K. Vegso, V. Nadazdy, P. Siffalovic, M. Jergel, M. Omastova, E. Majkova, *Mater. Today Commun.* **2023**, *36*, 106700. <https://doi.org/10.1016/j.mtcomm.2023.106700>.
6. D. Menzel, A. Al-Ashouri, A. Tejada, I. Levine, J. A. Guerra, B. Rech, S. Albrecht, L. Korte, *Adv. Energy Mater.* **2022**, *12*, 2201109. <https://doi.org/10.1002/aenm.202201109>.
7. L. Bertoluzzi, C. Boyd, N. Rolston, J. Xu, R. Prasanna, B. C. O’Regan, M. D. McGehee, *Joule* **2020**, *4*, 109. <https://doi.org/10.1016/j.joule.2019.10.003>.
